# Low testosterone in ApoE/LDL receptor double-knockout mice is associated with rarefied testicular capillaries together with fewer and smaller Leydig cells

**DOI:** 10.1038/s41598-018-23631-9

**Published:** 2018-04-03

**Authors:** Kai Steinfeld, Daniela Beyer, Christian Mühlfeld, Andrea Mietens, Gerrit Eichner, Bora Altinkilic, Marian Kampschulte, Qingkui Jiang, Gabriele A. Krombach, Thomas Linn, Wolfgang Weidner, Ralf Middendorff

**Affiliations:** 1 Department of Urology, Pediatric Urology and Andrology, Justus-Liebig-University Giessen, Giessen, Germany; 2 Institute of Anatomy and Cell Biology, Justus-Liebig-University Giessen, Giessen, Germany; 3 Institute of Mathematics, Justus-Liebig-University Giessen, Giessen, Germany; 4 Department of Radiology, Justus-Liebig-University Giessen, Giessen, Germany; 5 Centre of Internal Medicine, Justus-Liebig-University Giessen, Giessen, Germany; 6 Institute of Functional and Applied Anatomy, Hannover Medical School, Hannover, Germany

## Abstract

The testis as a site for atherosclerotic changes has so far attracted little attention. We used the apolipoprotein E (ApoE)/low density lipoprotein (LDL) receptor deficient mouse model (KO) for atherosclerosis (20, 40, 60 and 87-week-old) to investigate whether Leydig cells or the capillary network are responsible for reduced serum testosterone levels previously observed in extreme ages of this model. In KO mice, overall testosterone levels were reduced whereas the adrenal gland-specific corticosterone was increased excluding a general defect of steroid hormone production. In addition to micro-CT investigations for bigger vessels, stereology revealed a reduction of capillary length, volume and surface area suggesting capillary rarefaction as a factor for diminished testosterone. Stereological analyses of interstitial cells demonstrated significantly reduced Leydig cell numbers and size. These structural changes in the testis occurred on an inflammatory background revealed by qPCR. Reduced litter size of the KO mice suggests hypo- or infertility as a consequence of the testicular defects. Our data suggest reduced testosterone levels in this atherosclerosis model might be explained by both, rarefication of the capillary network and reduced Leydig cell number and size. Thus, this study calls for specific treatment of male infertility induced by microvascular damage through hypercholesterolemia and atherosclerosis.

## Introduction

Decline of androgen blood levels, dysfunctional sperm, and histologic changes, such as focal atrophy of seminiferous tubules and reduction of tubular diameter, are considered signs of male hypogonadism^1^. Focal tubular sclerosis was also reported in association with atherosclerotic lesions of interstitial testicular vessels^2^.

For a given organ, a healthy capillary vascular bed with its regular architecture is the prerequisite for adequate perfusion and function of this organ. The appearance of a regular testicular microvasculature was described previously for different species^3–6^. In man, early investigations of undescended testes linked reduced blood perfusion to atrophy and hypoplasia of the organ^7^. Reduction of blood flow due to decreased diameter of microvascular lumen in testes was suggested as a possible cause for degenerative changes of germ cells, oligospermia in humans^8^ and age-dependent disturbances of spermatogenesis^9,10^. A more recent study demonstrated that testicular vasculature might also be important for germ cell defects found in Klinefelter syndrome^11^.

Testosterone deficiency was reported together with signs of atherosclerosis in epidemiological studies, however, there is currently no evidence for a causal relationship^12–14^. Vice versa, testosterone administration was reported to inhibit atherosclerosis^15,16^ implying that lower testosterone levels promote atherosclerosis and coronary artery disease^17^.

Atherosclerotic plaques develop by retention of lipids in large- and medium-sized arteries as a consequence of shear stress to the vascular wall initiating platelet aggregation and blood coagulation. Plaques decrease downstream blood flow by reduction of the vascular lumen and thus have a negative impact on effective local blood circulation. Although evidence supports local immune processes induced by atherosclerotic plaques, there is considerable data to suggest an additional systemic immune response characterized by proinflammatory cytokines, such as TNFα released by endothelial cells, macrophages, and platelets^18–20^.

The relationship between vasculature and morphological alterations in different organs was investigated more systematically in a mouse model of atherosclerosis (ApoE^−/−^/LDL receptor^−/−^ mice) displaying enhanced plasma cholesterol levels. In this model a lipoprotein pattern resulting in hypercholesterolemia is caused by targeted mutations of both apolipoprotein E (ApoE) and the low-density lipoprotein (LDL) receptor^21^. ApoE, mainly synthesized in the liver, transports lipoproteins and cholesterol into the lymphatic system and is a ligand of the LDL receptor family. In the ApoE^−/−^/LDL receptor^−/−^ model, fatty streaks typical of advanced atherosclerotic lesions were observed in the proximal aorta and renal artery with increasing age^22^. In general, the degree of atherosclerosis detectable in wild-type (WT) mice is less pronounced compared to men. However, homozygous ApoE^−/−^/LDL receptor^−/−^ mice develop atherosclerotic alterations more similar to humans^23^. Disturbances of vasculature in ApoE and ApoE/LDL receptor deficient mice, respectively, were found not only in aortic root but also in lung^24^, heart^24,25^, kidney^22^ and bladder^26^.

In the testis, alterations of the vascular network accessible by micro-CT were found in very old ApoE/LDL receptor deficient mice on Western diet, high in calories from fat, and associated to a decreased quality of spermatogenesis with mixed atrophy, reduced testis volume, sperm count and serum testosterone levels^27^. However, this study could neither discriminate age-dependent changes from atherosclerosis nor resolve the actual capillary network which is beyond the resolution of micro-CT. Testicular capillaries are essential for the blood supply of seminiferous tubules and therefore are an important factor for maintenance of male fertility. Leydig cell integrity may also be disturbed by insufficient capillary perfusion or direct effects due to the specific characteristics of the ApoE^−/−^/LDL receptor^−/−^ mouse model. Testicular capillaries and Leydig cells have not yet been addressed in this model until now.

Here we investigated serum testosterone levels of 20, 40, 60 and 87-week-old ApoE^−/−^/LDL receptor^−/−^ mice and WT controls, both on standard chow. It was tested whether potential changes of hormone values could be due to mutation-induced disturbances in the capillary microcirculation or Leydig cell number and size using a design-based stereology system in addition to micro-CT analyses of the testes.

## Material and Methods

### Experimental design

This animal study was conducted in accordance to the German Animal Welfare Act and approved by the regional council of Giessen, Germany (GI 20/25A 43/2011). Male ApoE^−/−^/LDL receptor^−/−^ (KO) mice and male C57BL/6 J (wild-type, WT) mice (Charles River, Sulzbach, Germany) as controls were fed standard chow ad libitum throughout the study. They were euthanized with CO_2_. In both groups, KO and WT mice, animals of 20, 40, 60 and 87 weeks of age were used. Each age group included 10 mice except the oldest group (87 weeks) with three KO mice and six WT mice.

From each animal blood was collected from the vena cava caudalis for determination of steroids.

The left ventricle was penetrated by a cannula and infused with heparinized saline (10 ml of 0.9% sodium chloride with 1000 IU Heparin)^27^.

When the venous effluent was free of blood, the fixation solution (1.5% glutaraldehyde and 1.5% paraformaldehyde in 0.15 M HEPES) was infused and one testis was removed for histological morphometry, the ventricle was purged again with heparinized saline, and infused with the lead-containing radiopaque polymer (Microfil_MV-122; Flow Tech, Carver, MA, USA) for micro-CT investigations and voxel-based morphometry as described previously^27^.

For analyses of inflammation-associated parameters by real-time PCR 37-week-old KO and WT mice (n = 10) on standard chow were used.

### Determination of steroids (gas chromatography-mass spectrometry)

The blood samples from mice of the different age groups were centrifuged and the separated serum was investigated by gas chromatography-mass spectrometry.

This method^28^, consisting of many sub-steps, includes over-night incubation of the probes, extraction of the steroids by Extrelut® columns (Merck, Darmstadt, Germany), filtration with Sephadex LH-20 columns (Amersham Pharmacia Biotech AB, Uppsala, Sweden) and subsequent derivatization with HFBA (heptafluorobutyric anhydride, Fluka, Sigma-Aldrich, Steinheim, Germany).

### Voxel-based morphometry (Micro-computed tomography)

The contrast agent-perfused testes of mice from the different age groups were scanned in a micro-computed tomograph (micro-CT; SkySkan 1072_80kV, Kontich, Belgium) to investigate testis volume and vessel volume.

The x-ray system consists of a microfocus tube (20–80 kVp) with a minimum spot size of 8 Im at 8 W. While the sample carrier rotates at 180° at its own axis the sample is irradiated by x-rays in cone-beam geometry to generate projection images^27^.

These digital images were reconstructed in cross-section data sets by modified Feldkamp-algorithm with isotropic voxel geometry, so 3D images were available for evaluation by analysis software (Analyze 9.0 Mayo Clinic, Rochester, MN, USA and CTAn, SkyScan, Kontich, Belgium) to investigate testis volume and vessel volume in the agent-perfused testes of WT and KO mice.

### Histological morphometry (stereology)

Stereology was used as a method that allows the quantification of three-dimensional parameters of a given organ from histological sections by superimposing appropriate counting test systems like point grid, line grid and counting frame, on randomly sampled organ sections^18,29,30^. For this, testes of 20, 40, 60 and 87-week-old mice were fixed by vascular perfusion with a mixture of 1.5% glutaraldehyde and 1.5% paraformaldehyde in 0.15 M HEPES buffer. The testis volume was estimated by fluid displacement^31^ and the testes were cut into cubic tissue pieces (approximately 1 mm³) from which a random selection was osmicated, stained en-bloc with uranyl acetate and finally embedded in epoxy resin (Agar 100 Resin Kit, Agar Scientific, Stansted Essex, UK). The samples were randomly dropped into gelatine capsules (one sample for each capsule) to ensure random tissue orientation^32^. After embedding, semi-thin sections (0.75 µm) were cut using an ultramicrotome and stained with methylene blue. This procedure is well known to allow identification of all testicular cell types under physiological and pathological conditions^33–35^. In most cases, five sections from different tissue blocks per testis and mouse were investigated at the age of 20, 40, 60 and 87 weeks using design-based stereology^18^. Specifically, the length, volume and surface area of the capillaries of the testes were estimated. For this purpose, fields of view were sampled by systematic uniform random sampling at a magnification of 400× (Suppl. Figure 1) and superimposed with appropriate test systems using an Olympus microscope equipped with a digital camera and connected to a computer with the newCAST software (Visiopharm, Horsholm, Denmark). The volume density of capillaries related to the testis was estimated by counting the number of test points hitting capillaries (P(cap)) and the number of test points hitting testis (P(testis)). The volume density is given by V_V_(cap/testis) = P(cap)/P(testis). The surface density of the luminal capillary endothelium was estimated by counting the number of intersections (l(cap)) of test line segments with the luminal endothelium and relating them to the number of endpoints of the line segments hitting testis (P(testis)). The surface density is given by S_V_(cap/testis) = 2*l/(l(p)*P(testis)) where l(p) is the length of an individual test line associated with one endpoint. The length density was estimated by counting the number of capillary profiles (Q(cap)) within a counting frame of known area (A(CF)) and the number of corner points of the counting frame hitting testis P(testis). The length density is given by L_V_(cap/testis) = 2Q/(a/p*P(testis)) where a/p is the area of the counting frame associated with one corner point. The total capillary volume, surface area and length were calculated by multiplying the density with the testis volume^36^.

To investigate Leydig cell number and size, epoxy resin blocs were cut in 1 µm semi-thin sections. To exclude duplicate counting of Leydig cells, the first and fourth section of a consecutive row of sections were placed on a glass slide to generate a physical disector^32^ with a disector height of 3 µm (d). After staining, these paired sections were subjected to a systematic uniform random sampling and an unbiased counting frame with a known area (A(CF)) was projected on the fields of view. The number of Leydig cells was estimated by the disector principle, i.e. a cell was counted if the cell was visible in one section but not the other one (Q^−^). In addition, the number of corner points of the counting frame hitting the interstitium (P(interstitium)) was counted to estimate the reference volume. The number of Leydig cells in relation to the interstitium is given by N_v_(LC/interstitium) = Q^−^ /((A(CF)/4)*P(interstitium)*d). Based on this, the number of Leydig cells in relation to the entire testis is given by L(LC, testis) = N_v_(LC/interstitium)*V_v_(interstitium/testis)*V(testis). The disector was not only used for counting of cell number but also for sampling of the cells for volume estimation using the rotator^37^, i.e. if a cell was counted its volume was estimated by the rotator. From the resulting individual cell volumes, the number-weighted mean volume was calculated as the arithmetic mean of the measured cell volumes.

### Determination of litter size

The number of pups per litter and relative frequency of litter sizes were retrospectively evaluated in KO mice compared to WT mice. For that we analyzed data of 109 litter of KO and 43 litter of WT mice, every litter developed from 1:1-matings of young females and old males.

### Real-time reverse transcription-polymerase chain (qRT-PCR) reaction

Total RNA from testis and epididymal fat was extracted using RNeasy Mini Kit according to the manufacturer’s protocol. Total RNA (1.0 μg) was used as template for cDNA synthesis by using MMLV RT (Invitrogen). For quantitative comparisons, cDNA samples were analyzed by real-time PCR using the IQ SYBR Green Supermix on the StepOne Plus real-time polymerase chain reaction system (Applied Biosystems). Primer sequences are given in the supplement (Suppl. Table).

### Statistical analysis

The statistical analysis was performed using the statistics software R, version 3.0.2^38^. A value of p < 0.05 was considered significant.

### Steroids and morphometric parameters

For each parameter (steroids, testis volume, testis:body weight ratio, vessels volume, absolute and relative capillary length, volume and surface area and also Leydig cell number and size) the potential influence of age (in groups of 20, 40, 60 and 87 weeks) and mouse-strain (KO vs. WT) was investigated by a two-factorial ANOVA (with interaction-effect of age groups and mouse strain). When indicated this was complemented by multiple mean comparisons of the two mouse strains for the four age groups and simultaneous confidence intervals for the respective differences in mean values^39,40^.

The joint (multivariate) distribution of families of capillary parameters (absolute capillary length, volume and surface area and relative capillary length, volume and surface area) was analyzed using a MANOVA (with the same two factors as above and their interaction).

Three-dimensional scatter plots and parallel plots were used as exploratory tools for the presentation of the multivariate distribution of the mentioned parameter families. Parallel plots attempt to display relationships between several metric variables (measured as a multivariate observation on the same individual) by connecting their one-dimensional (=univariate) scatter plots. To this end, the measurement scales of the variables were placed parallel to each other, and the values that belong to the same multivariate observation, i.e. individual, were linked by line segments across the univariate scatter plots. This allows us to identify the values that constitute a multivariate observation by tracing the respective connected line segments (“polylines”) through the univariate scatter plots^41–43^. Since detection of relationships between variables is of primary interest, and to simplify the presentation, scale divisions or units are not provided, but only their orientation (by indicating their minimum and maximum).

### Litter size

The distribution of litter size of the two mouse strains was compared using Fisher´s exact test and the arithmetic means of those distributions were compared with Welch´s two-sided two-sample t-test (which was deemed admissible due to the fact that the large total sample size of 152 litter justifies the use of asymptotic properties of the test statistic).

### Inflammation-associated parameters

Data are given as mean values ± SEM, with *n* denoting the number of independent experiments unless otherwise indicated. A Welch´s two-sided two-sample t-test was used to determine statistical significance for qRT-PCR results.

### Material and data availability

ApoE^−/−^/LDL receptor^−/−^ mice (Stock No: 012307) and C57BL/6J (Stock No: 000664) are now available from Jackson Laboratory (Bar Harbor, ME, USA).

The datasets analysed in this study can be made available from the corresponding author on reasonable request.

## Results

### Serum hormone levels

Reduced serum testosterone levels had been described in very old ApoE^−/−^/LDL receptor^−/−^ mice under additional Western diet^27^. To investigate whether testosterone serum levels are also reduced independently of ageing and dietary effects we compared WT and KO mice between 20 and 87 weeks on standard chow.

**Figure 1:**
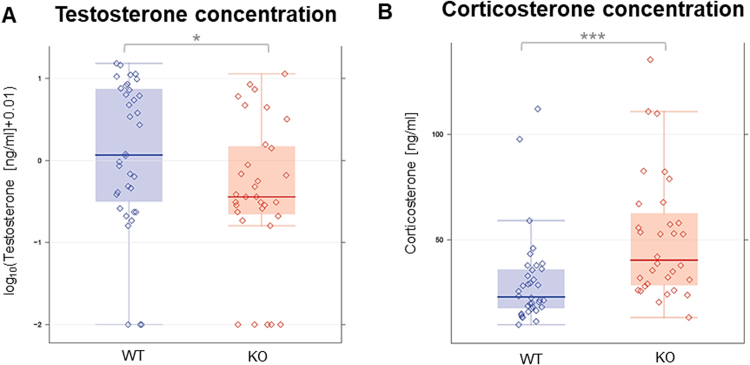
Serum levels of testosterone presented as log_10_(x + 0.01) (**A**) and corticosterone (**B**) in 20, 40, 60, 87-week-old KO and WT mice. KO mice showed a significant decrease of testosterone and a significant increase of corticosterone compared to WT mice. *p < 0.05, **p < 0.01, ***p < 0.001.  KO raw data;  WT raw data;  KO median;  WT median;  highest value still within 1.5 times the interquartile range of the third quartile of KO.  highest value still within 1.5 times the interquartile range of the third quartile of WT.  lowest value still within 1.5 times the interquartile range of the first quartile of KO.  lowest value still within 1.5 times the interquartile range of the first quartile of WT.  values between first and third quartile of KO data (indicating the middle 50% and the interquartile range).  values between first and third quartile of WT data (indicating the middle 50% and the interquartile range).

In KO mice, the overall serum testosterone levels were reduced as opposed to WT mice (p < 0.05) (Fig. 1A). Additional investigation of corticosterone levels, revealing higher values in KO mice (p < 0.001, Fig. 1B), suggested the absence of general defects in the production of steroid hormones.

### Testis volume, testis:body weight ratio and micro-CT-derived vessel volume

**Figure 2:**
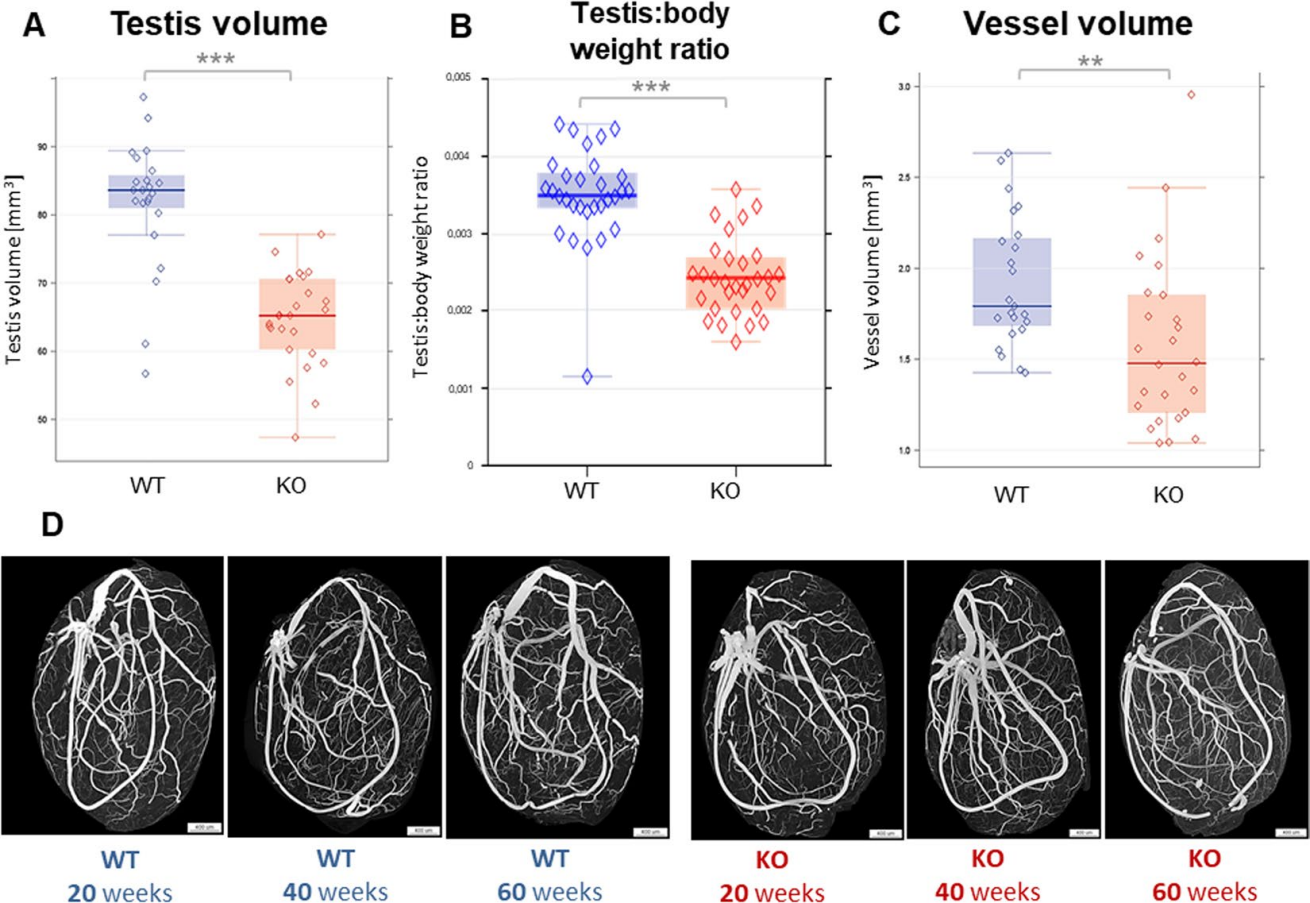
(**A**–**C**) Testis volume (**A**), testis:body weight ratio (**B**) and testicular vessel volume (**C**) analyzed by micro-CT investigation (**B**) showed a significant decrease of all parameters in 20, 40, 60-week-old KO compared to WT mice. *p < 0.05, **p < 0.01, ***p < 0.001.  KO raw data;  WT raw data; 3  KO median;  WT median.  highest value still within 1.5 times the interquartile range of the third quartile of KO.  highest value still within 1.5 times the interquartile range of the third quartile of WT.  lowest value still within 1.5 times the interquartile range of the first quartile of KO.  lowest value still within 1.5 times the interquartile range of the first quartile of WT.   values between first and third quartile of KO data (indicating the middle 50% and the interquartile range).  values between first and third quartile of WT data (indicating the middle 50% and the interquartile range). (**D**) Representative micro-CT images of different age groups from WT and KO mice.

In our efforts to evaluate possible reasons for the reduced testosterone levels in ApoE^−/−^/LDL receptor^−/−^ mice, we first analysed whether decreased testis and vessel volume, previously observed in old KO mice under Western diet, are inherent characteristics of this atherosclerosis model. Indeed, without particular diet overall testicular (Fig. 2A) volume was significantly reduced (p < 0.001) in KO compared to WT mice. The testis:body weight ratio (Fig. 2B) was also significantly reduced (p < 0.001).

Likewise, overall vessel volume (Fig. 2C) derived from micro-CT investigations (Fig. 2D) showed a significant decrease (p < 0.01) in KO mice.

These results confirm that atherosclerosis is associated to a reduced size of the testis in line with reduced vessel volume. However, the resolution of micro-CT only gives insufficient information on micro-vessels with a diameter of less than 15 μm.

### Capillary parameters

**Figure 3:**
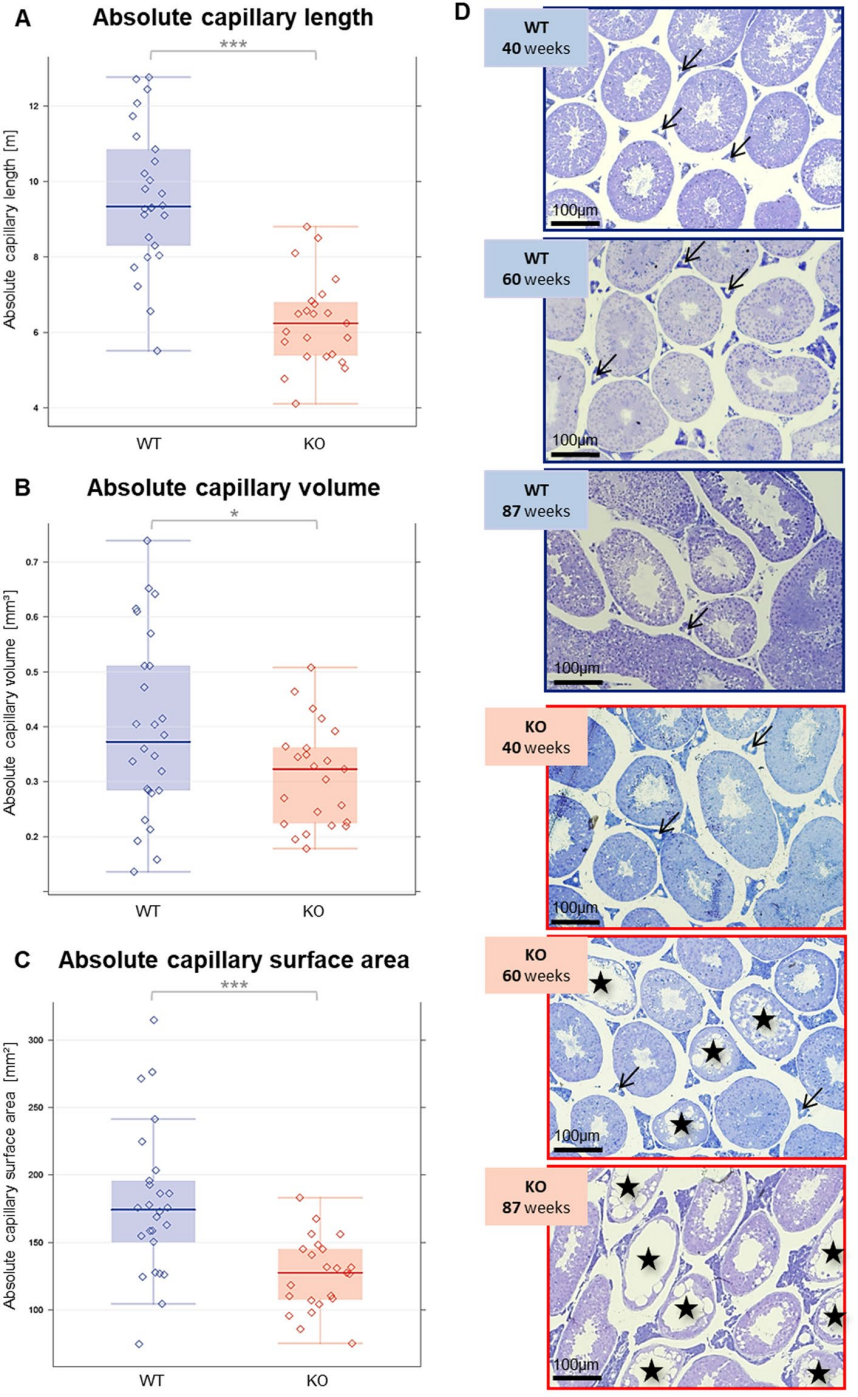
(**A**–**C**) Absolute capillary length (**A**), capillary volume (**B**) and capillary surface area (**C**) in 40, 60, 87-week-old KO and WT mice (*p < 0.05, **p < 0.01, ***p < 0.001) as determined by stereology.  KO raw data;  WT raw data;  KO median;   WT median;  highest value still within 1.5 times the interquartile range of the third quartile of KO;  highest value still within 1.5 times the interquartile range of the third quartile of WT;  lowest value still within 1.5 times the interquartile range of the first quartile of KO;  lowest value still within 1.5 times the interquartile range of the first quartile of WT;   values between first and third quartile of KO data (indicating the middle 50% and the interquartile range);   values between first and third quartile of WT data (indicating the middle 50% and the interquartile range);  **↓** Capillaries; ӿ seminiferous tubules showing disturbed spermatogenesis. (**D**) Representative semithin sections from WT and KO testes of the different age groups are shown as an overview. (Arrows indicate examples of capillaries, asterisks mark tubules with disturbed spermatogenesis).

To evaluate possible disturbances of the testicular capillary system as a factor for the reduced serum testosterone levels, a designed-based stereology procedure in semi-thin sections of glutaraldehyde-perfused testes was applied for determination of absolute and relative amount of small blood vessels. Absolute capillary length (p < 0.001, Fig. 3A), volume (p < 0.05, Fig. 3B) and surface area (p < 0.001, Fig. 3C) were significantly reduced in the univariate analysis of KO versus WT mice after adjusting for age. Representative semi-thin sections of the different age groups are shown in Fig. 3D as an overview. Higher magnification of such sections was used for stereological analyses (Suppl. Figure 1).

**Figure 4:**
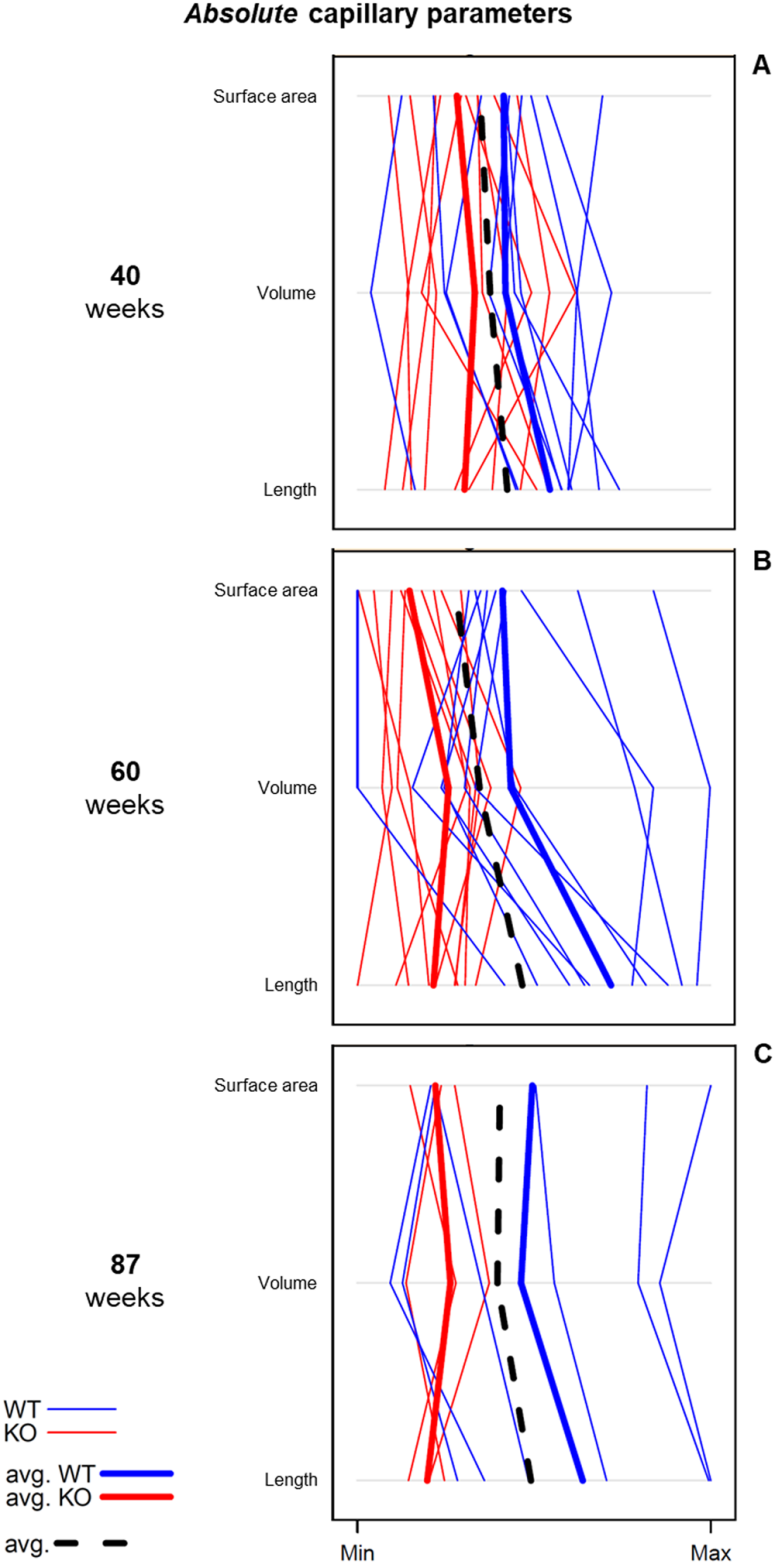
Parallel plots as exploratory tools for the presentation of the multivariate distribution of the parameter families (absolute capillary length, volume and surface derived from stereology) of KO (red lines) and WT mice (blue lines) at the age of 40 (**A**), 60 (**B**) and 87 (**C**) weeks. Parallel plots display relationships between several metric variables (measured as a multivariate observation on the same individual) by connecting their one-dimensional (=univariate) scatter plots. The measurement scales of the variables are placed parallel to each other, and the values that belong to the same multivariate observation, i.e. individual, are linked by line segments across the univariate scatter plots. Since detection of relationships between variables is of primary interest, and to simplify the presentation, scale divisions or units are not provided, but only their orientation (by indicating their minimum and maximum).

In addition to the aforementioned univariate analysis, the three capillary parameters were analyzed jointly (multivariately) to account for their interdependency reflecting the complexity of the microvascular network. Figure 4 shows parallel plots for the three absolute capillary parameters for all combinations of age group (40, 60, 87 weeks in separate panels) and mouse strain (WT, KO; colour-coded within each age-panel). They visualize the significant main effect of strain (p < 0.001) after accounting for age which is readily identifiable in Fig. 4 from the fact that the thick red average KO polylines are consistent to the left of the thick blue WT ones.

Interestingly, the overlap of KO and WT polylines decreased from 40 over 60 to 87-week-old mice (being responsible for the respective increase in differences between the average polyline-trends of WT and KO), suggesting an age-dependent reduction of micro-vessels in ApoE^−/−^/LDL receptor^−/−^ mice. Furthermore, for WT mice the variability of capillary parameters appeared to increase from 40-week-old to older (60, 87 weeks) animals (reflected in the slightly wider spread of the polylines in the higher age groups).

**Figure 5:**
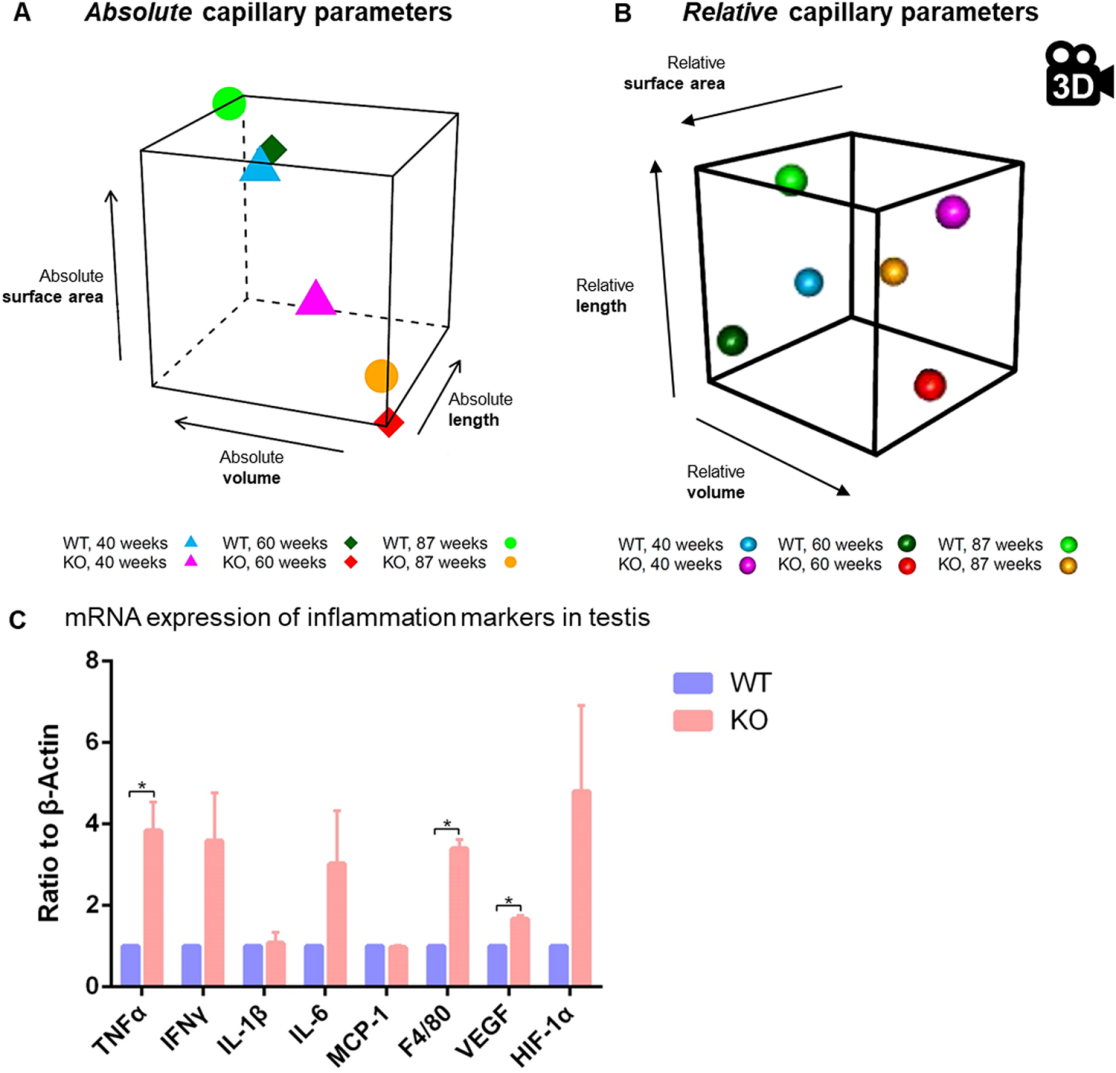
(**A**,**B**) View of three-dimensional distribution of the mean values of stereological capillary parameters of KO (magenta, red, yellow) and WT mice (blue, dark green, green) in the capillary length (y-axis), volume (z-axis) and surface area (x-axis) of absolute capillary parameters (**A**) and relative capillary parameters (**B**, snapshot of the Suppl. Movie) at the age of 40 (triangles, magenta/blue), 60 (quadrangle, red/dark green) and 87 (dot, yellow/green) weeks. (**C**) mRNA expression of inflammation markers in testis of KO compared to WT revealed by qRT-PCR studies. *p < 0.05.

The multivariate mean values of absolute capillary parameters for all combinations of age group and strain are also presented as three-dimensional scatter plots in Fig. 5A. Herein, the significant main effect of strain becomes easily apparent through the fact that the group of mean values of WT mice (for the three age groups) are localized in the rear upper range of the depicted cube while the respective group of KO mean values are in the front lower part.

Likewise, the multivariate mean values of the relative (i.e., compared to testis volume) capillary parameters are shown in Fig. 5B. Although those measurements do not show a distribution pattern as striking as in the absolute case, both main effects also turned out to be significant here: strain (p = 0.0194) and age (p = 0.0237). This becomes apparent when viewing the animated cube in the movie (Suppl. Movie) which allows appreciation from all directions of the three-dimensional space.

These structural changes were accompanied by increased gene expression levels of pro-inflammatory cytokines in KO opposed to WT mice with TNFα and VEGF reaching a level of statistical significance. Interestingly, the macrophage-specific marker F4/80 was upregulated (Fig. 5C).

In summary, these findings suggest that testicular microcirculatory changes occur also at the capillary level in this atherosclerosis model and could contribute to the observed reduced serum testosterone levels.

### Leydig cell number and Leydig cell size

Next, it was tested, whether potential changes of Leydig cell number and size could also represent a factor for reduced testosterone levels in the KO mice.

**Figure 6:**
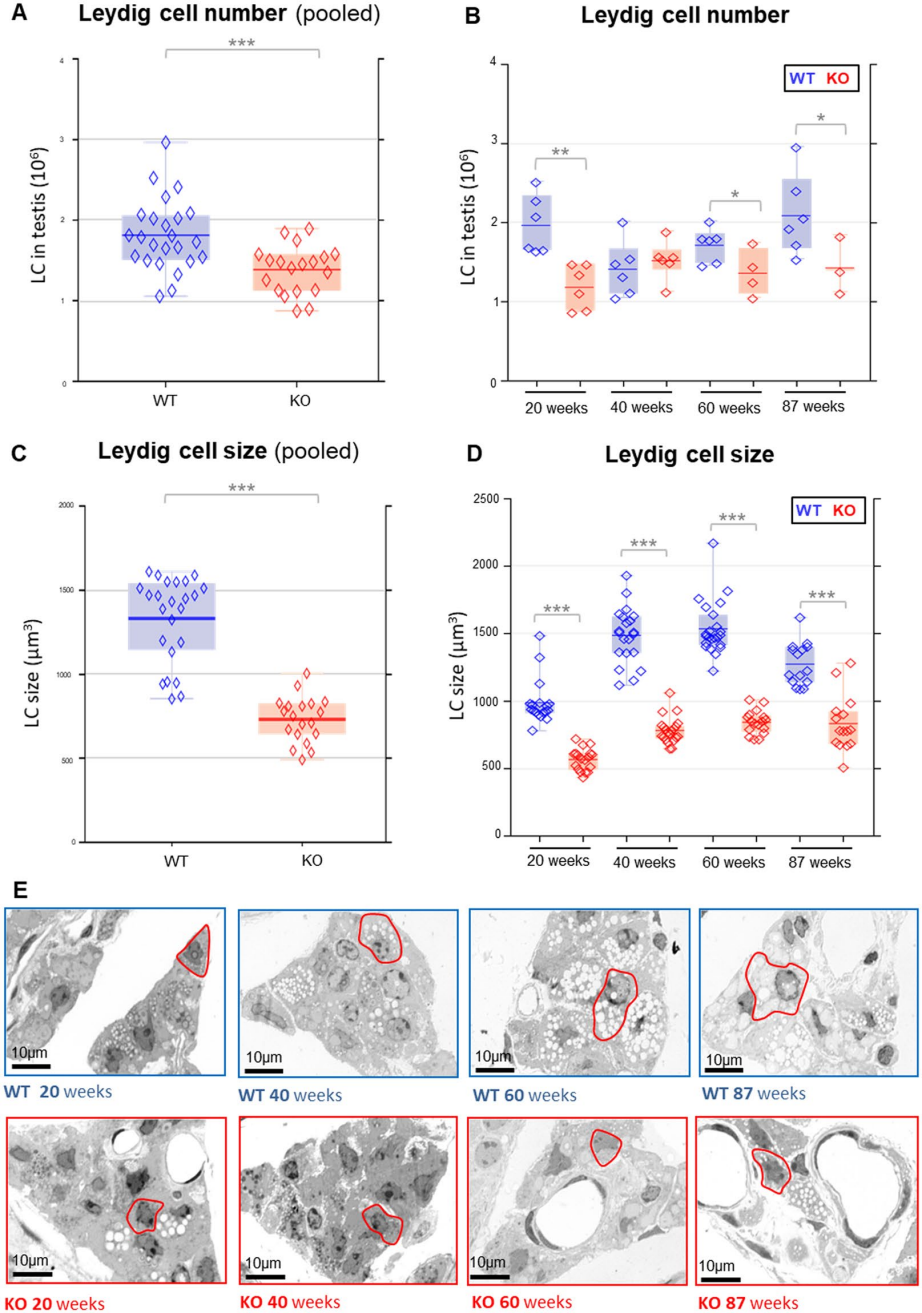
(**A**–**D**) Leydig cell number (**A** and **B**) and size (**C** and **D**) in KO and WT mice analyzed by stereological investigations on semithin sections. (**A,C**) Leydig cell number (**A**) and size (**C**) of data from pooled age groups (20, 40, 60, 87 weeks). (**B**,**D**) Leydig cell number (**B**) and size (**D**) from the individual age groups. *p < 0.05, **p < 0.01, ***p < 0.001.  KO raw data;  WT raw data;  KO median;  WT median.  highest value still within 1.5 times the interquartile range of the third quartile of KO.  highest value still within 1.5 times the interquartile range of the third quartile of WT.  lowest value still within 1.5 times the interquartile range of the first quartile of KO.  lowest value still within 1.5 times the interquartile range of the first quartile of WT.  values between first and third quartile of KO data (indicating the middle 50% and the interquartile range).  values between first and third quartile of WT data (indicating the middle 50% and the interquartile range). (**E**) Histological images of the testicular interstitial tissue of the different age groups in WT and KO mice (examples of Leydig cells are encircled in red).

Comparing KO and WT mice by our stereological approach, pooled data of all age groups (20, 40, 60, 87 weeks) showed a significantly reduced Leydig cell number in ApoE^−/−^/LDL receptor^−/−^ mice (p < 0.001) (Fig. 6A). Analyzing the Leydig cell number in the different age groups revealed a decreased number of Leydig cells in 20, 60 and 87-week-old mice (Fig. 6B).

Leydig cell size was significantly (p < 0.001) smaller in KO mice when comparing data from pooled age groups (20, 40, 60, 87 weeks) (Fig. 6C). Reduced Leydig cell size was found in each age group in KO mice (Fig. 6D). With 732.07 µm^3^, the mean KO Leydig cell was only about half the size of the WT Leydig cell with 1331.84 µm^3^. This reduction of the cell size was also readily visible in the histological pictures (Fig. 6E, representative Leydig cells are encircled in red).

### Litter size

Finally, the observed testicular changes led us to a retrospective analysis of litter size to obtain first information on fertility of ApoE^−/−^/LDL receptor^−/−^ mice. In agreement with the outcomes of hormonal, vascular and microvascular parameters congenic homozygous back-crossings of young females and old males exhibited a significant difference to the litter size distribution of WT mice (p = 0.0117), and in particular a significantly different mean litter size (p < 0.001) (Suppl. Figure 2). The average KO litter size was 1.6 animals smaller (with a 95% confidence interval of [0.7, 2.5] for this difference).

## Discussion

In this study, we found reduced serum testosterone levels in ApoE^−/−^/LDL receptor^−/−^ mice compared to wild-type controls in age-matched groups from 20 to 87 weeks on standard chow. In our efforts to evaluate reasons for this hormone deficiency, we could identify a rarefaction of the capillary system and a diminished number and size of Leydig cells as possible causal factors.

It is shown systematically for the first time that disturbances of one of the main functions of the testis, i.e. the supply of testosterone for the body, is connected to a reduction of the aggregated capillary parameters (capillary length, volume and surface). The testicular capillary network surrounds the seminiferous tubules and runs in the interstitial tissue of the testis comprising scarce connective tissue and the Leydig cells^44–46^. Especially, in each interstitial Leydig cell cluster capillaries are obligatory components running in the midst of the cluster in close proximity to all cells^46^ thus enabling the endocrine function of the Leydig cells.

Capillary networks are differently influenced by atherosclerosis depending on nutritional conditions of endothelia. The testicular capillary network which is rarefied in ApoE^−/−^/LDL receptor^−/−^ mice may affect Leydig cells and their function at different levels. By limiting the supply of nutrients, general aspects of cell function may be jeopardized. Testosterone production, the Leydig cell specific function, depends on the local supply and uptake of cholesterol as a substrate^47,48^. Similarly, LH as the main stimulator of Leydig cell function is delivered to its target cell by capillary perfusion^47^ and in a model of Sertoli cell ablation, vascular disturbances prevented LH from reaching its target^49^.

In line with an adequate afflux of nutrients, substrates and stimulants, the Leydig cell also requires a sufficient oxygen supply to match the metabolic demand for testosterone synthesis. Interestingly, HIF1alpha is constitutively expressed in Leydig cells even under normoxic conditions^50,51^ supporting that the Leydig cell functions at the brink to hypoxia. In humans chronically exposed to high altitude thus hypobaric hypoxia, serum testosterone levels were significantly reduced^52^.

Hypoxic conditions may be balanced by formation of new micro-vessels, this has been shown e.g. in the aortic wall^53^. Endothelial cells initiate vessel formation and arteriosclerotic disease has been shown to interfere with endothelial regenerative function in various organs. In ApoE^−/−^ mice with chronically elevated cholesterol levels, endothelial barrier and repair function has been compromised after transient cerebral artery occlusion and VEGF-induced capillary formation of brain endothelial cells was attenuated^54^. Similarly, in a model of hindlimb ischemia abundant cholesterol inhibited endothelial proliferation and migration^55^. In the kidney, cortical microvascular remodeling was concomitant with increased intrarenal protein expression of MCP-1 and TNFα^56^ indicating associated inflammation. Increases of inflammatory cytokines TNFα and interleukin-6 were reported in regard to endothelial damage and are considered features of both endothelial dysfunction and early stages of plaque formation. Various markers for inflammation, endothelial dysfunction and plaque formation are therefore of interest and in our study, we have clearly identified increased inflammatory cytokine gene expression in ApoE^−/−^/LDL receptor^−/−^ testis as compared to wild-type testis.

In atherosclerosis, vascular function is compromised by oxidized LDL which induces endothelial dysfunction and injury, not only in atherosclerotic plaques of large arteries, but also in micro-vessels^57^, this is in agreement with the testicular microvascular changes observed in the present study. Arteriosclerotic disease may prevent adequate formation of new micro-vessels in the testis and result in the observed rarefication of the capillary network in our ApoE^−/−^/LDL receptor^−/−^ model.

Adequate capillary perfusion in the testis is necessary for efflux of steroids to the general circulation. Rarefication of the capillary network in our ApoE^−/−^/LDL receptor^−/−^ model reduces the surface area available for transendothelial exchange and thus likely limits testosterone transfer into the blood stream which results in lowered serum testosterone levels as also seen in a model of Sertoli cell ablation with consecutive vascular disturbances^49^. With limited perfusion of the testis, the local supply of sex hormone binding globulin (SHBG), the plasma transport protein for testosterone, may also be reduced and contribute to lower serum testosterone levels as suggested before in a xxy mouse model^58^.

Similarly, the rarefied capillary network may interfere with removal of waste products and cause oxidative stress in the testis. Reactive oxygen species (ROS) like H_2_O_2_ or advanced glycation end products (AGEs) have been reported to interfere with testosterone production^59,60^.

In addition to reduced serum testosterone levels we also found a reduced number and size of Leydig cells in the ApoE^−/−^/LDL receptor^−/−^ testis. Changes of Leydig cell parameters in the ApoE^−/−^/LDL receptor^−/−^ mouse could be due to indirect (vasculature-mediated) or direct effects on the Leydig cells or a combination of both.

The Leydig cell number of our control (wild type) mice is in agreement with previous findings^61,62^ indicating that atherosclerotic changes in ApoE^−/−^/LDL receptor^−/−^ mice cause a reduction of Leydig cell number.

Reduced Leydig cell size in ApoE^−/−^/LDL receptor^−/−^ mice might reflect reduced (i) volume of smooth endoplasmic reticulum, previously shown to correlate with lower testosterone levels^63^, and (ii) fewer mitochondria as found in conditions of oxidative stress^64^.

However, it is unlikely that LDL receptor mutation especially in Leydig cells represents the main cause for reduced Leydig cell number and size. In contrast to diminished testosterone levels, serum concentrations of another steroid hormone, corticosterone, were not reduced in KO mice, but instead, significantly elevated. It is conceivable that corticosterone release was stimulated by inflammatory cytokines and nutritional overflow which in turn accelerates the development of atherosclerotic changes in ApoE-deficient mice^65–67^.

Studies describing reduced total Leydig cell volume in rats exposed to hypobaric hypoxia^68^, could argue for vasculature-mediated aspects of Leydig cell changes.

It is also conceivable that the reduced Leydig cell population is due to the contribution of the testicular capillary network to the regeneration of Leydig cells. Pericytes located within the capillary walls have been shown to serve as Leydig progenitor cells during postnatal development and to regenerate the Leydig cell population after ablation by EDS^69^. Rarefication of the capillary network as observed in the ApoE^−/−^/LDL receptor^−/−^ model would diminish the availability of progenitors and thus impair regeneration potential.

Since ApoE^−/−^/LDL receptor^−/−^ mice exhibit mutations of both the receptor and its ligand they are considered an appropriate model to investigate atherosclerosis without atherogenic diet^23^. As opposed to our previous study utilizing Western diet protocol^27^, in the present study mice were administered regular balanced rodent chow to discriminate the mutant phenotype from dietary effects. In addition, the experimental design of this study included defined age groups (20, 40, 60 and 87 weeks). The members in younger groups outnumbered oldest because some mice died before the age of 87 weeks. High death rate was described in ApoE-deficient mice before^70,71^. In this study KO mice were compared to WT mice at exactly the same age. Thus, animals with severest atherosclerosis might have escaped on time analysis resulting in negative experimental bias and rather underestimate changes in the capillary parameters.

Against this background it is noteworthy that our stereological investigations in ApoE^−/−^/LDL receptor^−/−^ mice also revealed a contribution of age to the reduction of capillary parameters. They decreased from 40 over 60 to 87-week-old mice. In addition, very young KO mice still showed normal testosterone levels indicating again that steroid synthesis was functional in spite of LDL receptor mutation.

It is not clear whether disturbances of spermatogenesis described in old ApoE^−/−^/LDL receptor^−/−^ mice^27^ and ApoE^−/−^ mice^72^ on Western diet are predominantly due indirectly to Leydig cell / testosterone deficits or directly to rarefication of testicular vasculature. Since peritubular capillaries with their close proximity to undifferentiated spermatogonia were described as vascular niche for germ cells^73^ a negative effect on spermatogenesis may arise from capillary reduction. The relevance of intact testicular vasculature^34^ and perfusion^74^ for spermatogenesis was suggested before. Reduced testicular blood flow was shown to induce hypoxic conditions which result in degeneration of germinal epithelium with disturbed spermatogenesis^75,76^. Similarly, hypobaric hypoxia in the rat led to increased apoptosis of spermatogonia and spermatocytes^76^.

The number of pups per litter was significantly reduced in KO mice, suggesting a direct relationship between disturbed blood supply and male hypogonadism. Interestingly, ageing alone had been shown not to reduce litter size despite of its negative effects on sperm number^77,78^.

Although female mutants are known to be less affected by atherosclerosis, our data do not allow complete exclusion of female fertility disturbances as well^66,79^.

Our data offer new insights into atherosclerotic disease by adding the testis as a hitherto underappreciated organ where one of its integral functions, providing the body with testosterone, is disturbed. Data showed that rarefaction of the capillary microvasculature in the testis together with reduced number and size of testosterone-producing Leydig cells might be causal factors. Thus, atherosclerosis contributes to the pathological changes of the testis and needs to be integrated into the management of male infertility.

## Electronic supplementary material

Supplementary Movie

Supplementary Information
