# Supplementary material for: Low testosterone in ApoE/LDL receptor double-knockout mice is associated with rarefied testicular capillaries together with fewer and smaller Leydig cells
Source: Sci Rep. 2018 Apr 3;8:5424. doi: 10.1038/s41598-018-23631-9 (PMC5882941; doi:10.1038/s41598-018-23631-9)
Supplement: Supplementary file 2 — Supplementary Information [file 41598_2018_23631_MOESM2_ESM.doc]

**Low testosterone in ApoE/LDL receptor double-knockout mice is associated with rarefied testicular capillaries together with fewer and smaller Leydig cells**

Kai Steinfeld^1,2 #^, Daniela Beyer^2 #^, Christian Mühlfeld^6^, Andrea Mietens^2^, Gerrit Eichner^3^, Bora Altinkilic^1^, Marian Kampschulte^4^, Qingkui Jiang^5^, Gabriele A. Krombach^4^, Thomas Linn^5^, Wolfgang Weidner^1*^, Ralf Middendorff^2*^

^1^Department of Urology, Pediatric Urology and Andrology, ^2^Institute of Anatomy and Cell Biology, ^3^Institute of Mathematics, ^4^Department of Radiology, ^5^Centre of Internal Medicine, Justus Liebig University Giessen, Germany, ^6^Institute of Functional and Applied Anatomy, Hannover Medical School, Germany

^*^corresponding author

^#^equal contribution

**Supplementary Info**

**Suppl. Movie** Animated three-dimensional distribution of relative capillary parameters (length, volume and surface area). Modified snapshot of this movie is shown in Fig. 5B.

**Suppl. Fig. 1.** Representative semi-thin section in the magnification used for stereological analyses of blood vessels. Capillary (C), artery (A), vascular smooth muscle cell (red arrow), Leydig cell (LC). The stereology-specific test systems are superimposed on this image: line grid (black), point grid (green), counting frame (green / red).


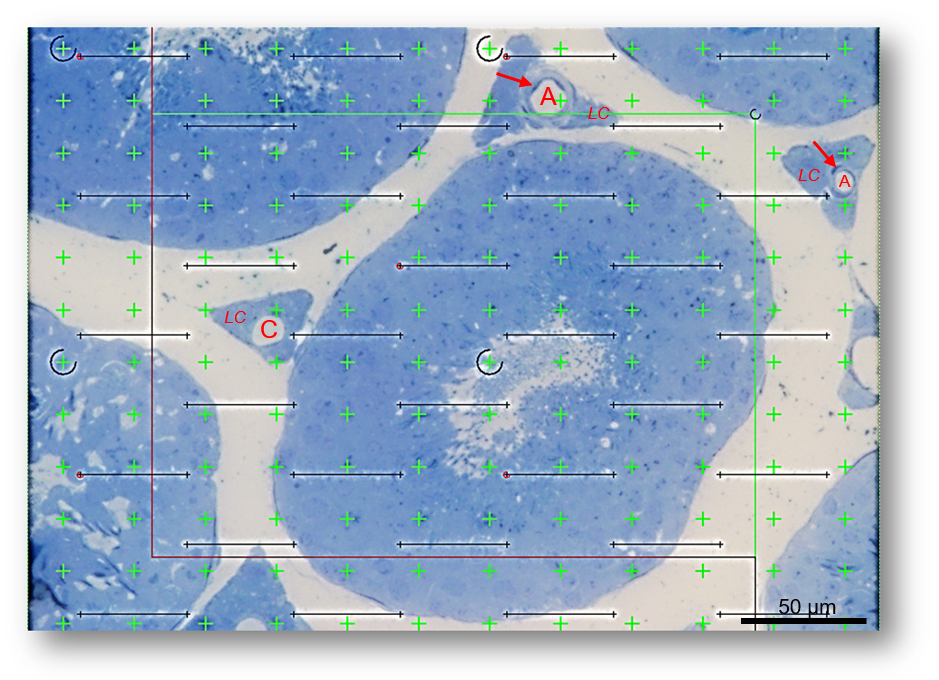


**Suppl. Fig. 2.** Retrospective comparison of litter size between KO (red) and WT mice (blue). In A the distributions of observed absolute litter sizes for KO (red, 109 litter) and WT (blue, 43 litter) mice are shown. In B the distribution of relative frequencies (in %) of observed absolute litter sizes is shown.

◊ KO raw data; ◊ WT raw data; ▬ KO median; ▬ WT median; ┬ highest value still within 1.5 times the interquartile range of the third quartile of KO; ┬ highest value still within 1.5 times the interquartile range of the third quartile of WT; ┴ lowest value still within 1.5 times the interquartile range of the first quartile of KO; ┴ lowest value still within 1.5 times the interquartile range of the first quartile of WT; █ values between first and third quartile of KO data (indicating the middle 50% and the interquartile range); █ values between first and third quartile of WT data (indicating the middle 50% and the interquartile range).


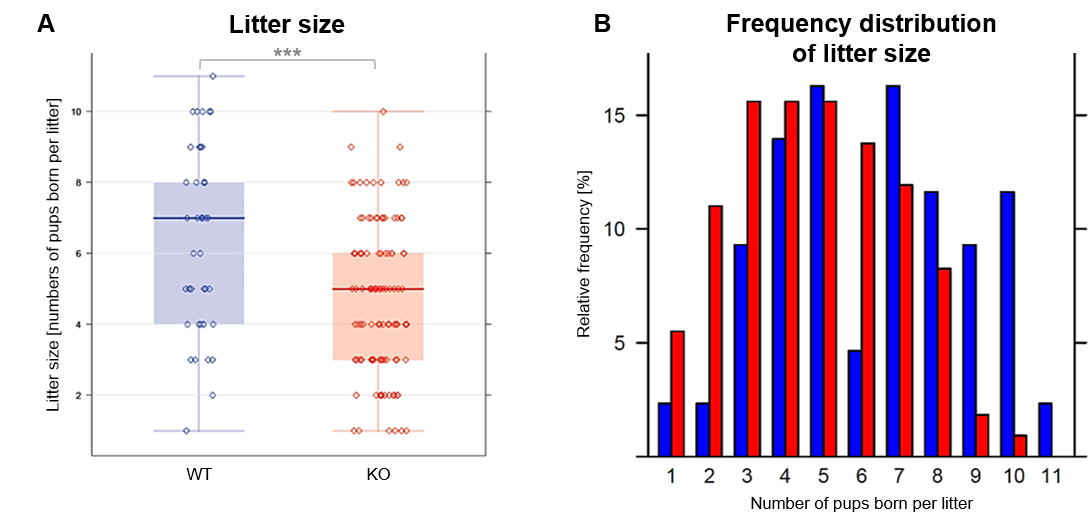


**Suppl. Table** Primer sequences

| GAPDH | fwd | 5’-TCCATGCCATGACTGCCACTC-3’ |
| --- | --- | --- |
|  | rev | 5’-TGACCTTGCCCACAGCCTTG-3’ |
| TNFα | fwd | 5’-CATCTTCTCAAAATTCGAGTGACAA-3’ |
|  | rev | 5’-TGGGAGTAGACAAGGTACAACCC-3’ |
| IFN-γ | fwd | 5’-TCAAGTGGCATAGATGTGGAAGAA-3’ |
|  | rev | 5’-TGGCTCTGCAGGATTTTCATG-3’ |
| IL-1β | fwd | 5’-AGGTCGCTCAGGGTCACAAG-3’ |
|  | rev | 5’-GTGCTGCCTAATGTCCCCTTGAATC-3’ |
| IL-6 | fwd | 5’-GACAACTTTGGCATTGTGG-3’ |
|  | rev | 5’-ATGCAGGGATGATGTTCTG-3’ |
| MCP-1 | fwd | 5’-CTGGATCGGAACCAAATGAG-3’ |
|  | rev | 5’-CGGGTCAACTTCACATTCAA-3’ |
| F4/80 | fwd | 5’-CTTTGGCTATGGGCTTCCAGTC-3’ |
|  | rev | 5’-GCAAGGAGGACAGAGTTTATCGTG-3’ |
| VEGF | fwd | 5’-GCACTGGACCCTGGCTTTAC-3’ |
|  | rev | 5’-AATGCTTTCTCCGCTCTGA-3’ |
| HIF-1α | fwd | 5’ -TGCTTGGTGCTGATTTGTGA-3’ |
|  | rev | 5’ -GGTCAGATGATCAGAGTCCA-3’ |
